# A Taxonomy Proposal for Types of Interactions of Language and Place-Value Processing in Multi-Digit Numbers

**DOI:** 10.3389/fpsyg.2018.01024

**Published:** 2018-06-25

**Authors:** Julia Bahnmueller, Hans-Christoph Nuerk, Korbinian Moeller

**Affiliations:** ^1^Neuro-cognitive Plasticity Laboratory, Leibniz-Institut für Wissensmedien, Tübingen, Germany; ^2^Department of Psychology, Eberhard Karls University of Tübingen, Tübingen, Germany; ^3^LEAD Graduate School and Research Network, University of Tübingen, Tübingen, Germany

**Keywords:** linguistic influences, numerical processing, place-value processing, multi-digit numbers, number word inversion

## Abstract

Research on associations between language and number processing has seen growing interest in the last years – in particular with respect to place-value processing in multi-digit numbers. Recently, Dowker and Nuerk (2016) proposed a taxonomy of linguistic influences on number processing. However, this taxonomy does not address the generality or specificity of linguistic influences across different levels of number processing. In contrast, Nuerk et al. (2015) proposed different levels of place-value processing in multi-digit numbers. However, the authors did not specify if and how linguistic factors influence these levels of place-value processing. The present perspective aims at addressing this conceptual gap by suggesting an integrated taxonomy representing how different linguistic factors may influence different levels of place-value processing. We show that some effects of different linguistic levels have already been observed on different levels of place-value processing. Moreover, while some linguistic influences (e.g., lexical influences) have been studied for all levels of place-value processing, other influences have been studied for only one level or even none. Beyond categorizing existing research, we argue that the explicit consideration of research gaps may inspire new research paradigms complementing the picture of language influences on place-value processing. We conclude by outlining the importance of a differential approach for levels of both linguistic and number processing to evaluate linguistic obstacles and facilitators of different languages and their relevance for numerical development.

## Introduction

Linguistic or language influences have seen growing research interest in the area of number processing and particularly with regard to place-value processing in multi-digit numbers. A systematic classification of levels of linguistic influences as well as their direction was recently proposed by Dowker and Nuerk (2016). However, the primary focus in numerical cognition research was (and still often is) on single-digit number processing. This may be problematic, because findings and conclusions obtained from research on single-digit numbers cannot simply be transferred to multi-digit numbers (cf. Nuerk et al., 2011). For instance, the majority of difficulties in numerical development specifically relate to numbers and procedures beyond the single-digit number range (e.g., Zuber et al., 2009; for transcoding). For multi-digit Arabic numbers, one specifically crucial concept that needs to be acquired and understood is their place-value structuring principle. This principle reflects that the magnitude of a digit within the digit string (and consequently also of the overall number) can only be derived if spatial information regarding the position of digits within the digit string is considered. In particular, the spatial sequence of digits determines the value of a specific digit in descending powers of the base 10 from left to right (e.g., 4242 = {4}× 10^3^+{2}× 10^2^ +{4} × 10^1^+{2}× 10^0^). Importantly, different levels of processing place-value information were specified (Nuerk et al., 2015). Thus, not only are there different linguistic levels affecting (multi-digit) number processing, but there are also different levels of place-value processing that can and should be distinguished.

Therefore, we argue that it is necessary to specify levels both of linguistic influences and place-value processing which are addressed in a specific paradigm to be able to distinguish and classify conceptually (dis)similar mechanisms underlying associations of language and place-value processing in multi-digit symbolic numbers. Such a classification comes with the opportunity to evaluate whether every linguistic influence is indeed relevant to each level of place-value processing and/or whether linguistic influences affect (only) specific levels of place-value processing. As a starting point, we suggest integrating the previously proposed taxonomy on linguistic factors influencing number processing by Dowker and Nuerk (2016) and the classification of different levels of place-value processing by Nuerk et al. (2015).

## Linguistic Levels Interacting With (Multi-Digit) Number Processing

Large-scale cross-cultural studies, like TIMSS or PISA (e.g., Mullis et al., 2012; OECD, 2014) showed repeatedly that mathematical competences of children vary considerably between countries. One of the main and consistent findings is the superiority in mathematic performance of countries such as China, Japan, or Korea, also called the “Chinese number advantage” (e.g., Miura et al., 1993; Miura and Okamoto, 2003). Over and above educational systems and socio-economic factors (e.g., Towse and Saxton, 1998; Miller et al., 2005; Ngan Ng and Rao, 2010), linguistic specificities have been suggested to impact mathematical performance in general and place-value processing in particular. To specifically classify associations between linguistic specificities and number processing, Dowker and Nuerk (2016) recently introduced a taxonomy of six different linguistic levels: (A) lexical, (B) visuo-spatial orthographic (C) phonological (D) semantic (E) conceptual, and (F) syntactic.

The *lexical* level is the most widely investigated and is concerned with specificities on the number word level with respect to the transparency of power (e.g., in Chinese, power is explicit in number symbols and words: 42 = 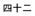 = 4 10 2) and transparency of order (e.g., the inversion of number words: in German the number word corresponding to 42 is zweiundvierzig, literally two and forty). The *visuo-spatial orthographic* level is not a typical linguistic category in most of the linguistic literature. However, this level includes effects of reading and writing direction and reading behavior that have been shown to heavily influence spatial-numerical processing (e.g., determining the direction of spatial numerical associations, Shaki et al., 2009). The *phonological* level summarizes effects of phonological processes and/or deficits as well as effects related to verbal working memory. Influences on the phonological level are, for instance, reflected by effects of concurrent articulation on specific aspects of number processing – indicating their reliance on verbal/phonological processing (e.g., Moeller et al., 2011). The *semantic* level is concerned with influences and characteristics of words (other than number words) and symbols that convey numerical meaning (e.g., more, less, buy, sell, +, -, cm, m). In this context, Shikhare et al. (2015) showed, for instance, that numerical estimation and comparison strategies as well as quantifier semantics determine the processing of proportional quantifiers (e.g., “few”, “many”, and “some”). Numerical processing is also influenced by certain linguistic *concepts* such as, for instance, linguistic markedness [e.g., there are unmarked (even, right) and marked forms (odd, left) of most adjective pairs]. Here, the effect of linguistic markedness of response codes (MARC effect, Nuerk et al., 2004) describes the finding that responses are faster for congruent pairings (i.e., even number/right hand response, odd number/left hand response) than incongruent ones (i.e., even number/left hand response, odd number/right hand response). Finally, the *syntactic* level refers to influences of grammar resulting from, for instance, specificities of certain grammatical rules. In this context, grammatical number was found to support learning cardinality of small numbers using the give-N task which requires the processing of the respective magnitude information. Sarnecka et al. (2007) compared groups of three-year-olds speaking languages with (English, Russian) and without plural markings (Japanese) and showed that more English/Russian than Japanese children gave the correct number of items indicating that grammar may have facilitated the acquisition of number cardinality.

In sum, the above taxonomy illustrates that language may be associated with number processing at different linguistic levels. As such, the term association is used intentionally to underline the potential bidirectionality of influences. Importantly, linguistic levels do not have to be task-relevant but might still influence the way we process numbers in a highly automatic yet implicit manner (as for reading direction and spatial-numerical associations; Shaki et al., 2009). Finally, more than one linguistic level may be associated with number processing: Moeller et al. (2015a) showed both lexical (inversion) as well as visuo-spatial orthographic (reading direction) influences on performance in a multi-digit number comparison task. However, place-value processing in multi-digit numbers is not unidimensional either. Both task requirements and processing characteristics that are specific to a respective task play a crucial role, and thus, linguistic levels might be equally important for some but not all tasks.

## Place-Value Processing Levels in Multi-Digit Numbers

Regarding multi-digit numbers, Nuerk et al. (2015) suggested three different levels of place-value processing to classify different tasks according to processing requirements: (1) place identification, (92) place-value activation, and (3) place-value computation.

*Place identification* is suggested to be an early and very basic requirement of virtually all tasks involving multi-digit numbers. This process is required for correctly identifying the position of a single digit within the digit string (e.g., tens and units positions in two-digit numbers) without the necessity of further processing the magnitude of these digits. An exemplary task involving place identification is transcoding of multi-digit numbers (i.e., writing numbers to dictation). With respect to transcoding, Nuerk and colleagues suggest that although magnitude information (by means of place-value activation) may be processed in addition to place identification, magnitude processing is not necessary (see also Cipolotti and Butterworth, 1995; Barrouillet et al., 2004).

In contrast to transcoding, other tasks such as number magnitude comparison require the *activation of place-value information*, which means that each symbol (digit) is associated with a specific position (place). Without place-value activation, the “Which number is larger?” question simply cannot be answered.

Finally, some tasks additionally require *place-value computation* in terms of changes or updates of value and/or place. For example, to correctly execute a carry operation in an addition task, the decade digit of the unit sum needs to be added to the sum of the decade digits to correctly solve the task (e.g., for 28+17, 8+7 = **1**5, and thus the sum of the decade digits needs to be updated accordingly, i.e., 2+1+**1** = 4). As such, carry problems are more difficult than non-carry problems (e.g., Deschuyteneer et al., 2005).

Taken together, there are different levels of linguistic influences on number processing and different levels of place-value processing for multi-digit numbers. Therefore, we suggest classifying any interaction of language and place-value processing in multi-digit numbers according to both, the level of linguistic influence and place-value processing.

## Integrating Levels of Linguistic Influences and Place-Value Processing

**FIGURE 1:**
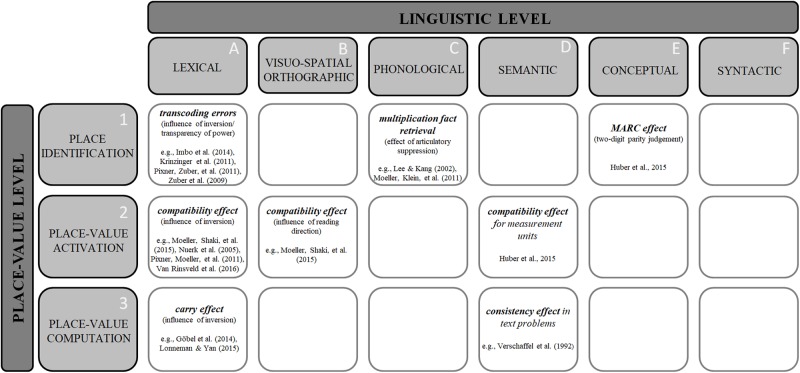
Schematic illustration of a selection of previously observed associations of language and multi-digit numbers differentiated by both the classification of levels of linguistic influences on number processing (cf. Dowker and Nuerk, 2016) and levels of place-value processing (cf. Nuerk et al., 2015).

Classifying processes underlying different tasks and manipulations according to both linguistic and place-value processing levels results in a grid as depicted in **Figure 1**. Therein, each cell describes the association of one specific level of linguistic influences (A to F) with one specific level of place-value processing (1 to 3). It becomes evident that some associations have already been studied quite extensively, while others have been addressed only rarely or not at all so far.

A closer look at the studies investigating linguistic influences on (multi-digit) number processing indicates that two major approaches can be distinguished: first, cross-linguistic studies, comparing number processing effects across different languages or cultures, and second, linguistic manipulations that vary specific linguistic features within one language and evaluate differential effects on number processing.

### Cross-Linguistic Approaches

Interestingly, the lexical and the visuo-spatial orthographic levels are dominated by cross-linguistic approaches focusing on number processing effects that are sensitive to influences of specific aspects of language systems. On the *lexical level*, two important aspects that vary between number word systems have been shown to influence place-value processing: transparency of power and transparency of order. Detrimental influences of nontransparent number word systems were identified in a variety of tasks and paradigms on all three levels of place-value processing. Regarding the association of place identification and lexical influences (**Figure 1**, A1), transcoding performance was shown to be specifically vulnerable to inversion-related errors (i.e., writing down 45 when dictated 54, e.g., Zuber et al., 2009; Krinzinger et al., 2011; Pixner et al., 2011b; Imbo et al., 2014; for specific errors in Japanese, see Moeller et al., 2015b). Moreover, with respect to place-value activation (**Figure 1**, A2), specific differences between inverted and non-inverted languages were observed for the unit-decade compatibility effect in two-digit number magnitude comparison [i.e., compatible number pairs (32_57, 3 < 5 and 2 < 7) are responded to faster than incompatible pairs (37_62, 3 < 6 but 7 > 2); Nuerk et al., 2001]. For both children (Pixner et al., 2011a) and adults (Nuerk et al., 2005; Moeller et al., 2015a; but see Ganor-Stern and Tzelgov, 2011) it was found that interference due to the irrelevant unit digit is more pronounced for languages with an inverted number word system. Finally, lexical influences were also investigated at the level of place-value computation (**Figure 1**, A3). For instance, Göbel et al. (2014) observed that the carry effect was more pronounced in German- (inverted number words) than Italian-speaking children (no inversion; see also Colomé et al., 2010; Lonnemann and Yan, 2015).

Investigations of influences of reading/writing direction on number processing (reflecting the *visual-spatial orthographic level*) have their origin in the assumption of a mental number line on which numbers are arranged from left to right in ascending order. This metaphor indicates a close association of numbers and space. Evidence for this claim comes, for example, from the SNARC effect (Dehaene et al., 1993), showing that in Western cultures smaller numbers are usually associated with the left-hand side, whereas larger numbers are associated with the right-hand side (for visual-spatial orthographic influences on spatial-numerical associations see Göbel et al., 2011; but see, e.g., van Dijck and Fias, 2011 for a working memory account and Schroeder et al., 2017 for a multiple coding account on the SNARC-effect). Regarding multi-digit numbers and with respect to the level of place-value activation, Moeller et al. (2015a) considered both visual-spatial orthographic (reading direction) and lexical influences (inversion) in a quadrilingual cross-cultural study with German- and English-speaking adults (left-to-right reading languages with inverted and non-inverted number words, respectively) as well as Hebrew and Arabic speakers (right-to-left read languages with inverted and non-inverted number words, respectively; **Figure 1**, B2 and A2). Results indicated that compatibility effects were larger when the order of digits in symbolic Arabic notation did not match the order of tens and units in number words (i.e., German and Hebrew). Importantly, this study illustrates that levels of linguistic influences should not be considered in isolation because more than one linguistic level might actually impact number processing at the same time.

It is important to note that not every cross-linguistic study is also cross-cultural. First, samples can be chosen for which the cultural environment is held constant. For instance, Mark and Dowker (2015) investigated linguistic influences on mathematical development between language groups but within the same culture and educational system. In particular, Mark and Dowker (2015) compared children that spoke Chinese at home and learnt to count in Chinese at school to children that spoke Chinese at home and learnt to count in English at school. Therefore, major cultural discrepancies (e.g., educational system, cultural environment) were balanced between the two samples (for similar within-culture approaches see Dowker et al., 2008; Colomé et al., 2010; Pixner et al., 2011b; Imbo et al., 2014). Second, the investigation of bilingual speakers also allows for an investigation of cross-linguistic differences within one and the same culture (e.g., Macizo et al., 2010a,b; Macizo et al., 2011a,b; Van Rinsveld et al., 2016). Crucially, when investigating bilingual speakers not only differences between numerical processing in the respective languages but also potential cross-linguistic modulations can be evaluated [e.g., whether or not specificities of one language influence (numerical) processing in the other language; cf. Van Rinsveld et al., 2016]. Such cross-linguistic modulations might have important implications for practical interventions for bilingual speakers. In general, research on cross-linguistic, though not cross-cultural studies substantiated influences of lexical linguistic properties on all three levels of place-value processing.

### Language Manipulations

Instead of employing quasi-experimental designs comparing different language groups (or the same group in different language contexts) as described above, specific linguistic attributes may also be manipulated directly within one and the same language to identify additional interactions of linguistic and place-value processing levels. In particular, specific manipulations of phonological or semantic input as well as the consideration of specific linguistic concepts have already unraveled a variety of additional associations between levels of linguistic and place-value processing.

On the *phonological level*, for instance, Lee and Kang (2002) manipulated the availability of verbal information processing resources in multiplication and subtraction tasks and observed that concurrent articulation specifically reduced multiplication fact retrieval but not subtraction performance. This indicates that phonological processing of number words indeed affects place-value processing in multi-digit numbers differentially and even when no explicit magnitude processing is required to correctly solve the task (see also Moeller et al., 2011; **Figure 1**, C3).

Next to insights resulting from the manipulation of phonological processing resources, interactions of levels of linguistic influences and place-value processing were explored by considering stimuli that are semantically different from Arabic numbers or number words but still convey numerical meaning. By manipulating the semantic input, investigations on the *semantic level* allow for both an identification of effects resulting from specific word categories and/or for a generalization of number processing effects across different words/symbols. Referring to the former, for text problems it was observed that words associated with an addition procedure (e.g., “more,” “buy”) facilitated processing of text problems requiring additions whereas words associated with subtraction (e.g., “less,” “sell”) interfered with addition problem solving (e.g., Verschaffel et al., 1992; see also Daroczy et al., 2015 for a review on linguistic and numerical factors in text problems; **Figure 1**, D3). Referring to the latter, place-value processing also seems to be recycled for the processing of measurement units as typical effects observed for two-digit numbers (e.g., unit-decade compatibility effect) were also demonstrated for measurement units (Huber et al., 2015a; **Figure 1**, D2). Thus, these studies show that magnitude information is not only expressed and processed via Arabic digits and number words but also via other words and symbols which in some cases share processing specificities observed for place-value processing in multi-digit numbers.

Finally, there is first evidence for an interaction between linguistic aspects and place-identification on the *conceptual level*, specifically through manipulating the markedness of response codes. Huber et al. (2015b) investigated the MARC effect in a two-digit parity judgement task. A regular MARC effect was observed for both single- and (the unit digit of) two-digit numbers (**Figure 1**, E1). This suggests that the manipulation of specific linguistic concepts might interfere with place-value identification as well.

## Filling the Gaps: Inspiring Future Research

In addition to assigning different levels of linguistic and place-value processing to categorize existing research, a taxonomy may also inspire future research. For instance, addressing gaps at the visuo-spatial orthographic level, cross-cultural studies using a quadrilingual design comparable to the one used in Moeller et al. (2015a) might help to evaluate questions on the generality of visual-spatial orthographic influences across different place-value processing levels. Using, for instance, transcoding in children and/or addition tasks should allow for investigating influences on place-identification and the place-value manipulation level, respectively (i.e., **Figure 1**, B1 and B3).

Moreover, future research might also consider combining not only different linguistic levels but also different approaches (e.g., combining quasi-experimental and experimental designs). For example, it would be interesting to evaluate whether a linguistic effect determined on one linguistic level and in one language group generalizes to or differs from other language groups. On the syntactic level, for instance, effects of specific grammatical structures were found to influence processing of single-digit numbers (e.g., Sarnecka et al., 2007). However, these effects have not yet been investigated for multi-digit numbers. Potential syntactic effects on place-value processing might be investigated in language groups with differing ways of expressing grammatical number. For instance, in many languages, the singular is used in relation to one entity and plural for entities larger than one. In contrast, in Polish, the unit digits 2 to 4 are followed by plural verb forms whereas for the unit digits 1 and 5 to 9 singular is used. The same pattern holds for multi-digit numbers with the respective unit digits (e.g., 22 to 24 is followed by plural verb forms; 21 and 25 to 29 are followed by singular verb forms). In this context, the grammatical SNARC effect (i.e., singular associated with left and plural with right; Roettger and Domahs, 2015) might be investigated in a cross-linguistic study design to determine the language specificity of this syntactic effect and its potential generalizability to the multi-digit number range.

Finally, next to a broadening of our understanding of the generality and limits of interactions of linguistic and place-value processing levels, it will also be crucial to identify developmental trajectories of such interactions as well as their different effect sizes, i.e. their differential significance in practical contexts to be able to develop tailored types and time windows for potential interventions.

## Conclusion

Language considerably influences numerical cognition and development. Therefore, we suggest that it is important to understand the principles of such influences in any language. To foster such understanding, the goal of this article was to show that the general conceptualization, “language influences multi-digit number processing” captures neither the diversity of different levels of linguistic influences nor that of different levels of place-value processing. So far, a lot of research effort has been devoted to investigating prominent linguistic influences (mostly lexical), and has to a large part neglected others. We hope that this overview and taxonomy inspires researchers to study other linguistic influences on different levels of place-value processing as well to generate a more complete and differentiated picture of such interactions in the future. This will help us to better understand benefits and obstacles for numerical and arithmetic processing and learning in a given language and ultimately foster development and remediation tailored to each language background as well.

## Author Contributions

All authors contributed intellectually to the conceptualization and revision of this perspective paper and read and approved the submitted version. The initial draft was written by JB.

## Conflict of Interest Statement

The authors declare that the research was conducted in the absence of any commercial or financial relationships that could be construed as a potential conflict of interest.

## References

[B1] Baddeley A. Lewis V. Vallar G. (1984). Exploring the articulatory loop. *Q. J. Exp. Psychol. A* 36 233–252. 10.1080/14640748408402157 3369966

[B2] Barrouillet P. Camos V. Perruchet P. Seron X. (2004). ADAPT: a developmental, asemantic, and procedural model for transcoding from verbal to Arabic numerals. *Psychol. Rev.* 111 368–394. 10.1037/0033-295X.111.2.368 15065914

[B3] Cipolotti L. Butterworth B. (1995). Toward a multiroute model of number processing: impaired number transcoding with preserved calculation skills. *J. Exp. Psychol. Gen.* 124 375–390. 10.1037/0096-3445.124.4.375

[B4] Colomé À. Laka I. Sebastián-Gallés N. (2010). Language effects in addition: how you say it counts. *Q. J. Exp. Psychol.* 63 965–983. 10.1080/17470210903134377 19742389

[B5] Daroczy G. Wolska M. Meurers W. D. Nuerk H.-C. (2015). Word problems: a review of linguistic and numerical factors contributing to their difficulty. *Front. Psychol.* 6:348. 10.3389/fpsyg.2015.00348 25883575 PMC4381502

[B6] Dehaene S. Bossini S. Giraux P. (1993). The mental representation of parity and number magnitude. *J. Exp. Psychol. Gen.* 122 371–396. 10.1037/0096-3445.122.3.371

[B7] Deschuyteneer M. De Rammelaere S. Fias W. (2005). The addition of two-digit numbers: exploring carry versus no-carry problems. *Psychol. Sci.* 47 74–83.

[B8] Dowker A. Bala S. Lloyd D. (2008). Linguistic influences on mathematical development: how important is the transparency of the counting system? *Philos. Psychol.* 21 523–538. 10.1080/09515080802285511

[B9] Dowker A. Nuerk H.-C. (2016). Editorial: linguistic influences on mathematics. *Front. Psychol.* 7:1035. 10.3389/fpsyg.2016.01035 27462286 PMC4940406

[B10] Ganor-Stern D. Tzelgov J. (2011). Across-notation automatic processing of two-digit numbers. *Exp. Psychol.* 58 147–153. 10.1027/1618-3169/a000080 20705547

[B11] Göbel S. M. Moeller K. Pixner S. Kaufmann L. Nuerk H.-C. (2014). Language affects symbolic arithmetic in children: the case of number word inversion. *J. Exp. Child Psychol.* 119 17–25. 10.1016/j.jecp.2013.10.001 24269580

[B12] Göbel S. M. Shaki S. Fischer M. H. (2011). The cultural number line: a review of cultural and linguistic influences on the development of number processing. *J. Cross Cult. Psychol.* 42 543–565. 10.1177/0022022111406251

[B13] Huber S. Bahnmueller J. Klein E. Moeller K. (2015a). Testing a model of componential processing of multi-symbol numbers - Evidence from measurement units. *Psychon. Bull. Rev.* 22 1417–1423. 10.3758/s13423-015-0805-8 25651800

[B14] Huber S. Klein E. Graf M. Nuerk H.-C. Moeller K. Willmes K. (2015b). Embodied markedness of parity? Examining handedness effects on parity judgments. *Psychol. Res.* 79 963–977. 10.1007/s00426-014-0626-9 25394996

[B15] Imbo I. Vanden Bulcke C. De Brauwer J. Fias W. (2014). Sixty-four or four-and-sixty? The influence of language and working memory on children’s number transcoding. *Front. Psychol.* 5:313. 10.3389/fpsyg.2014.00313 24782811 PMC3990049

[B16] Krinzinger H. Gregoire J. Desoete A. Kaufmann L. Nuerk H.-C. Willmes K. (2011). Differential language effects on numerical skills in second grade. *J. Cross Cult. Psychol.* 42 614–629. 10.1177/0022022111406252 11699686

[B17] Lee K.-M. Kang S. (2002). Arithmetic operation and working memory: differential suppression in dual tasks. *Cognition* 83 B63–B68. 10.1016/S00100277(02)00010-0 11934408

[B18] Lonnemann J. Yan S. (2015). Does number word inversion affect arithmetic processes in adults? *Trends Neurosci. Educ.* 4 1–5. 10.1016/j.tine.2015.01.002

[B19] Macizo P. Herrera A. Paolieri D. Román P. (2010a). Is there cross-language modulation when bilinguals process number words? *Appl. Psycholinguist.* 31 651–669. 10.1017/S0142716410000184

[B20] Macizo P. Herrera A. Román P. Martín M. C. (2010b). Second language acquisition influences the processing of number words. *Procedia Soc. Behav. Sci.* 9 1128–1134. 10.1016/j.sbspro.2010.12.295

[B21] Macizo P. Herrera A. Román P. Martín M. C. (2011a). Proficiency in a second language influences the processing of number words. *J. Cogn. Psychol.* 23 915–921. 10.1080/20445911.2011.586626

[B22] Macizo P. Herrera A. Román P. Martín M. C. (2011b). The processing of two-digit numbers in bilinguals. *Br. J. Psychol.* 102 464–477. 10.1111/j.2044-8295.2010.02005.x 21752000

[B23] Mark W. Dowker A. (2015). Linguistic influence on mathematical development is specific rather than pervasive: revisiting the Chinese Number Advantage in Chinese and English children. *Front. Psychol.* 6:203. 10.3389/fpsyg.2015.00203 25767456 PMC4341514

[B24] Miller K. F. Kelly M. Zhou X. (2005). “Learning mathematics in China and the United States: cross-cultural insights into the nature and course of preschool mathematical development,” in *Handbook of Mathematical Cognition*, ed. Campbel J. I. D. (New York, NY: Psychology Press), 163–178.

[B25] Miura I. T. Okamoto Y. (2003). “Language supports for mathematics understanding and performance,” in *The Development of Arithmetic Concepts and Skills: Constructing Adaptive Expertise. Studies in Mathematics Thinking and Learning*, eds Baroody A. J. Dowker A. (Mahwah, NJ: Erlbaum), 229–242.

[B26] Miura I. T. Okamoto Y. Kim C. C. Steere M. Fayol M. (1993). First graders’ cognitive representation of number and understanding of place value: cross-national comparisons: France, Japan, Korea, Sweden, and the United States. *J. Educ. Psychol.* 85 24–30. 10.1037/0022-0663.85.1.24

[B27] Moeller K. Klein E. Fischer M. H. Nuerk H. Willmes K. (2011). Representation of multiplication facts - Evidence for partial verbal coding. *Behav. Brain Funct.* 7:25. 10.1186/1744-9081-7-25 21740568 PMC3148976

[B28] Moeller K. Shaki S. Göbel S. M. Nuerk H.-C. (2015a). Language influences number processing – A quadrilingual study. *Cognition* 136 150–155. 10.1016/j.cognition.2014.11.003 25497523

[B29] Moeller K. Zuber J. Olsen N. Nuerk H.-C. Willmes K. (2015b). Intransparent German number words complicate transcoding - A translingual comparison with Japanese. *Front. Psychol.* 6:740. 10.3389/fpsyg.2015.00740 26113827 PMC4462644

[B30] Mullis I. V. S. Martin M. O. Foy P. Arora A. (2012). *TIMSS 2011 International Results in Mathematics.* Chestnut Hill, MA: TIMSS & PIRLS International Study Center.

[B31] Ngan Ng S. S. Rao N. (2010). Chinese number words, culture, and mathematics learning. *Rev. Educ. Res.* 80 180–206. 10.3102/0034654310364764

[B32] Nuerk H.-C. Iversen W. Willmes K. (2004). Notational modulation of the SNARC and the MARC (linguistic markedness of response codes) effect. *Q. J. Exp. Psychol. A* 57 835–863. 10.1080/02724980343000512 15204120

[B33] Nuerk H.-C. Moeller K. Klein E. Willmes K. Fischer M. H. (2011). Extending the mental number line - A review of multi-digit number processing. *Z. Psychol.* 219 3–22. 10.1027/2151-2604/a000041

[B34] Nuerk H.-C. Moeller K. Willmes K. (2015). “Multi-digit number processing: overview, conceptual clarifications, and language influences,” in *The Oxford Handbook of Numerical Cognition*, eds Kadosh R. C. Dowker A. (Oxford: Oxford University Press), 106–139.

[B35] Nuerk H.-C. Weger U. Willmes K. (2001). Decade breaks in the mental number line? Putting the tens and units back in different bins. *Cognition* 82 B25–B33. 10.1016/S0010-0277(01)00142-1 11672709

[B36] Nuerk H.-C. Weger U. Willmes K. (2005). Language effects in magnitude comparison: small, but not irrelevant. *Brain Lang.* 92 262–277. 10.1016/j.bandl.2004.06.107 15721959

[B37] OECD. (2014). *PISA 2012 Results: What Students Know and Can do – Student Performance in Mathematics, Reading and Science*, Vol. I. Paris: PISA, OECD Publishing 10.1787/9789264201118-en

[B38] Pixner S. Moeller K. Heřmanová V. Nuerk H.-C. Kaufmann L. (2011a). Language effects on nonverbal number processing in first grade - A trilingual study. *J. Exp. Child Psychol.* 108 371–382. 10.1016/j.jecp.2010.09.002 21035126

[B39] Pixner S. Zuber J. Heřmanová V. Kaufmann L. Nuerk H.-C. Moeller K. (2011b). One language, two number-word systems and many problems: numerical cognition in the Czech language. *Res. Dev. Disabil.* 32 2683–2689. 10.1016/j.ridd.2011.06.004 21763104

[B40] Roettger T. B. Domahs F. (2015). Grammatical number elicits SNARC and MARC effects as a function of task demands. *Q. J. Exp. Psychol.* 68 1231–1248. 10.1080/17470218.2014.979843 25384199

[B41] Sarnecka B. W. Kamenskaya V. G. Yamana Y. Ogura T. Yudovina Y. B. (2007). From grammatical number to exact numbers: early meanings of ‘one’, ‘two’, and ‘three’ in English, Russian, and Japanese. *Cogn. Psychol.* 55 136–168. 10.1016/j.cogpsych.2006.09.001 17070794 PMC2322941

[B42] Schroeder P. A. Nuerk H.-C. Plewnia C. (2017). Switching between multiple codes of SNARC-like associations: two conceptual replication attempts with anodal tDCS in sham-controlled cross-over design. *Front. Neurosci.* 11:654. 10.3389/fnins.2017.00654 29217996 PMC5703834

[B43] Shaki S. Fischer M. H. Petrusic W. M. (2009). Reading habits for both words and numbers contribute to the SNARC effect. *Psychon. Bull. Rev.* 16 328–331. 10.3758/PBR.16.2.328 19293102

[B44] Shikhare S. Heim S. Klein E. Huber S. Willmes K. (2015). Processing of numerical and proportional quantifiers. *Cogn. Sci.* 39 1504–1536. 10.1111/cogs.12219 25631283

[B45] Towse J. N. Saxton M. (1998). “Mathematics across national boundaries: cultural and linguistic perspectives on numerical competence,” in *The Development of Mathematics Skills*, ed. Donlan C. (Hove: Psychology Press), 129–150.

[B46] van Dijck J. P. Fias W. (2011). A working memory account for spatial–numerical associations. *Cognition* 119 114–119. 10.1016/j.cognition.2010.12.013 21262509

[B47] Van Rinsveld A. Schiltz C. Landerl K. Brunner M. Ugen S. (2016). Speaking two languages with different number naming systems: what implications for magnitude judgments in bilinguals at different stages of language acquisition? *Cogn. Process.* 17 225–241. 10.1007/s10339-016-0762-9 27020298

[B48] Verschaffel L. De Corte E. Pauwels A. (1992). Solving compare problems: an eye movement test of Lewis and Mayer’s consistency hypothesis. *J. Educ. Psychol.* 84 85–94. 10.1037/0022-0663.84.1.85

[B49] Zuber J. Pixner S. Moeller K. Nuerk H.-C. (2009). On the language specificity of basic number processing: transcoding in a language with inversion and its relation to working memory capacity. *J. Exp. Child Psychol.* 102 60–77. 10.1016/j.jecp.2008.04.003 18499120

